# Buprenorphine to reverse respiratory depression from methadone overdose in opioid-dependent patients: a prospective randomized trial

**DOI:** 10.1186/s13054-020-2740-y

**Published:** 2020-02-07

**Authors:** Nasim Zamani, Nicholas A. Buckley, Hossein Hassanian-Moghaddam

**Affiliations:** 1 Social Determinant of Health Research Center, Shahid Beheshti University of Medical Sciences, Tehran, Iran; 2 Department of Clinical Toxicology, Loghman Hakim Hospital, Shahid Beheshti University of Medical Sciences, South Karegar Street, Tehran, Iran; 3 Toxicological Research Center, Shahid Beheshti University of Medical Sciences, Tehran, Iran; 4 Pharmacology, Faculty of Medicine and Health, University of Sydney, Sydney, Australia

**Keywords:** Opioid overdose, Antidote treatment, Methadone, Naloxone, Buprenorphine

## Abstract

**Background:**

Naloxone is the usual drug used in opioid-induced respiratory depression but it has a short half-life, precipitates withdrawal in dependent patients, and thus for persistent reversal of long-acting opioids has to be given by titrated doses and infusions. The partial agonist buprenorphine has a much longer duration of action and causes less severe withdrawal, but still should largely reverse respiratory depression induced by full agonist opioids. We aimed to compare the efficacy/safety of buprenorphine and naloxone in reversing respiratory depression in methadone-poisoned opioid-dependent patients.

**Methods:**

Patients with methadone-induced respiratory depression were randomized to receive naloxone (titrated doses), or lower or higher doses of buprenorphine (10 μg/kg or 15 μg/kg). The primary outcome was immediate reversal of respiratory depression. We also recorded acute opioid withdrawal, need for intubation/recurrent apnea, repeated doses of opioid antagonists, length of hospital stay, other morbidity, and mortality. The study was registered with the Iranian Registry of Clinical Trials (Trial ID: 18265; Approval code: IRCT2015011020624N1).

**Results:**

Eighty-five patients were randomized; 55/56 patients who received buprenorphine had rapid reversal of respiratory depression, which persisted for at least 12 h. Naloxone was effective in 28/29 patients, but often required very high titrated doses (thus delaying time to respond) and prolonged infusions. Intubation (8/29 vs 5/56) and opioid withdrawal (15/29 vs 7/56) were less common with buprenorphine. There were no serious complications or deaths in those receiving buprenorphine. The 15-μg/kg buprenorphine dose appeared to provide a longer duration of action, but precipitated withdrawal more frequently than the 10-μg/kg dose.

**Conclusion:**

Buprenorphine appears to be a safe and effective substitute for naloxone in overdosed opioid-dependent patients. Further studies are warranted to explore the optimal dosing strategy for buprenorphine to consistently maintain reversal of respiratory depression but not precipitate withdrawal.

**Trial registration number:**

IRCT2015011020624N1. Registered 30 September 2015.

**Electronic supplementary material:**

The online version of this article (10.1186/s13054-020-2740-y) contains supplementary material, which is available to authorized users.

## Introduction

Opioid overdose is a major cause of morbidity and mortality worldwide [1, 2]. The usual antidote treatment for opioid-induced respiratory depression is naloxone. Although safe in opioid-naive patients, naloxone may precipitate severe acute withdrawal, which may in turn cause severe complications including cardiac arrhythmias, and acute respiratory distress syndrome (ARDS) [3, 4]. Thus, doses are usually titrated to avoid giving excessive doses. The short half-life of naloxone (< 1 h) means that for longer-acting opioids repeated doses and infusions are often required, potentially for days [5].

The long-acting, high-potency, partial agonist buprenorphine might be expected to cause less withdrawal and persistent effective reversal, but there is little published evidence. There are anecdotes about successful bystander administration of buprenorphine/naloxone (Suboxone®) combinations [6, 7]. Effectiveness was also shown in an animal study and a single clinical case report [8, 9].

This pilot study provides further evidence on the efficacy and safety of buprenorphine in reversing of respiratory depression in methadone-poisoned opioid-dependent patients and compares this to standard naloxone therapy.

## Methods

### Study design and setting

This phase II open-label, parallel arm, controlled clinical trial was conducted from November 2015 to December 2018, in Loghman Hakim toxicological emergency department (ED), a very busy toxicology center that treats 24–28 thousand poisoning cases annually [10]. Written informed consent was taken from the patients’ next of kin before randomization in all cases. The protocol was approved by the ethics committee in Shahid Beheshti University of Medical Sciences and registered with the Iranian Registry of Clinical Trials (IRCT; Approval code: IRCT2015011020624 N1).

### Population

**Fig. 1:**
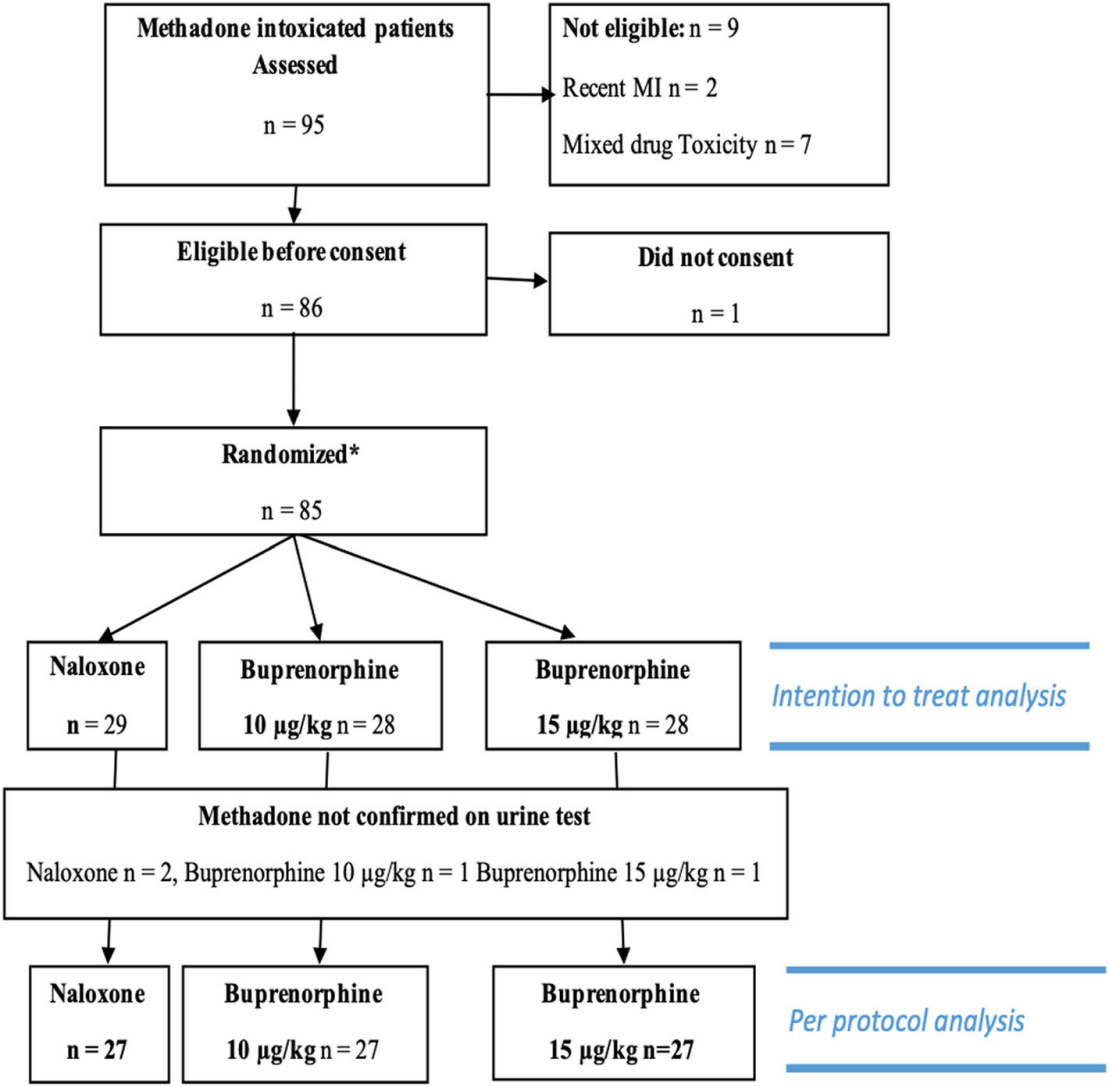
CONSORT diagram for patients’ allocation

We enrolled opioid-dependent methadone-poisoned patients who developed acute respiratory depression (cyanosis, decreased O_2_ saturation [less than 90% in the context of acute overdose] and RR < 12 per minute) and needed antidote treatment after presentation to hospital. All were believed to have overdosed on methadone, and urine tests were done for confirmation. Opioid dependence was indicated by chronic use of any opioid drug (tramadol, methadone, opium, codeine, etc.). Many patients had received an initial dose of naloxone before evaluation and subsequently redeveloped respiratory depression a short time later (this was not an exclusion). We excluded patients less than 15 years old, those with known co-ingestion of other drugs, those who had aspirated or needed intubation prior to evaluation, and those with known cardiovascular disease (Fig. 1).

We defined a per-protocol group (81/85) of those who had confirmed methadone on urine test and also had negative assays for non-opioid drugs. Urine and blood were assayed for 26 commonly ingested drugs (phenytoin, carbamazepine, phenobarbital, sodium valproate, diazepam, alprazolam, clonazepam, chlordiazepoxide, aspirin, acetaminophen, different tricyclic antidepressants, digoxin, iron, lithium, and ethanol).

### Interventions

Patients were randomly assigned into the three arms in the emergency department by a very rapid and simple bedside method. There was an opaque bag containing three identical balls with letter A, B, or C; the letters representing the three arms. Balls were mixed, and one was drawn to determine treatment.

Patients were bagged prior to antidote administration. All antidotes were given intravenously (IV). Group A received naloxone based on the titrated dosing recommendations in Goldfrank’s toxicologic emergencies [11]. These patients received 2 mg of naloxone if they developed apnea (respiratory rate [RR] less than 6 per minute) and 0.04 mg if they developed bradypnea (RR of 6 to 12 per minute) [11]. If the primary dose of naloxone was not sufficient to reverse the patient’s respiratory depression, the patients were given 0.4-, 2-, and finally 10-mg doses of naloxone in 2- to 3-min intervals to reverse respiratory depression [5, 11]. When these initial doses reversed the patient’s respiratory depression, they were put on a continuous infusion starting at a rate of two thirds of the effective bolus dose per hour, using an IV infusion pump. Subsequently, the naloxone infusion was tapered (halved roughly every 8 h) if the patient was completely symptom-free and venous blood gas analyses (VBGs) were normal. More rapid tapering was done if there is evidence of withdrawal. The infusion was ceased when the effective dose was less than 0.25 mg/h.

Groups B and C were given 10 μg/kg and 15 μg/kg of IV buprenorphine (slowly administered over 6 and 9 min, respectively). Patient’s weights were estimated. These two doses were based on unpublished experience and previous publications [8, 12–14].

For all three arms, if there was inadequate clinical response, the patient was intubated and this was considered as a treatment failure. Patients were to be observed for at least 24 h after the last dose of antidote.

### Primary and secondary outcomes

The primary outcome was the initial response to the administered antidote. The goal of antidote administration was re-institution of adequate spontaneous ventilation (increased respiratory rate and depth, increased oxygen saturation, resolution of cyanosis, and decline in PCO2 in the following VBGs). A partial (but adequate) response was indicated by an increase in respiratory rate, with oxygen saturation above 90% but residual sedation. A complete response was indicated if there was complete arousal to normal consciousness.

We also recorded re-development of respiratory depression/apnea over the admission. Although the primary outcome of response to the antidote was reached in all patients who were not intubated, some re-developed respiratory acidosis in the following hours. Persistent respiratory acidosis was defined as a pH of ≤ 7.30 and a pCO_2_ of ≥ 50 mmHg in spite of ongoing infusion of naloxone or after administration of the buprenorphine doses.

We recorded adverse outcomes including development of initial withdrawal syndrome (measured and quantified by Clinical Opiate Withdrawal Scale (COWS)) [15]. We also noted withdrawal syndromes occurring after subsequent antidote administration. We did not include later withdrawals which were attributed to opioid cessation. We also recorded medical complications including aspiration, acute respiratory distress syndrome (ARDS), myocardial infarction (MI), acute tubular necrosis (ATN), sepsis, disseminated intravascular coagulopathy (DIC), and death. ARDS was the most common adverse outcome and was diagnosed when there was an acute onset of bilateral pulmonary infiltrates, severe hypoxemia, and P/F ratio of less than 300 mmHg in the absence of evidence of cardiogenic pulmonary edema [16].

### Data collection

A pre-formatted form was used (by N.Z.) to record demographic and clinical variables. The variables evaluated included patient’s age, gender, and type of opioid dependency (to methadone, opium, tramadol, etc.), time elapsed between ingestion of methadone and hospital presentation, formulation (syrup versus tablet) and amount of the ingested methadone, Glasgow Coma Scale (GCS) on presentation, treatments given before presentation (if naloxone had been administered, naloxone dose, and development of withdrawal after its administration), treatments given in ED (naloxone or buprenorphine), their dose and response to them, infusion dose of naloxone (in group A) and the total time of its infusion, the dose of buprenorphine given (in groups B and C), development of withdrawal after administration of the antidote and its severity determined by COWS, need for sedation, need for intubation, total ICU and hospital stay, medical complications (ARDS, etc..), final outcome, and lab tests including 6-hourly venous blood gas analyses. Need for re-administration of naloxone or buprenorphine, and apnea after initial doses or during infusion were also recorded. Ward nurses were responsible for IV drug administration, measured vital signs, and recorded other events. There was no blinding of antidote allocation. Most data was prospectively collected; the formulation, amount, and time of ingestion of methadone were determined from the patient retrospectively.

### Data management and statistical analysis

Data was transcribed to a spreadsheet from the case-record forms for further analysis. Descriptive data are summarized with median [interquartile range] and range. Given the lack of previous data on expected response rates, no formal sample size calculation was performed. The target recruitment of 90 patients was based on typical phase II study strategies [17], and logistic considerations about the expected number of eligible patients over 3 years. As this was an exploratory proof of principle phase II study, we focused on comparisons via the “per protocol” analysis which excluded the small number of patients whose methadone ingestion was not confirmed. An “intention to treat” analysis was also performed (see Additional file 1).

Our main analysis combined the two buprenorphine groups and compared these to the naloxone group. We also compared outcomes in all three groups. We used Fisher’s exact test to compare proportions and the Mann-Whitney *U* test to compare continuous variables. We used graphical presentation and the log-rank test for time to event analysis (for recurrent respiratory depression). Alpha was set at 0.05, and we did not adjust for multiple comparisons. We used GraphPad Version 8.2 for all analyses.

## Results

A total of 95 adult opioid-dependent methadone-poisoned patients, who had respiratory depression on presentation or developed it during hospitalization, presented to Loghman Hakim toxicological ED during the study period and were considered for inclusion. Ten patients were excluded, and 85 patients were then randomly assigned to a treatment arm. In four patients, the urine test for substances of abuse did not test positive for methadone. Our per-protocol analysis focuses on the 81 remaining patients (CONSORT diagram; Fig. 1).

**Fig. 2:**
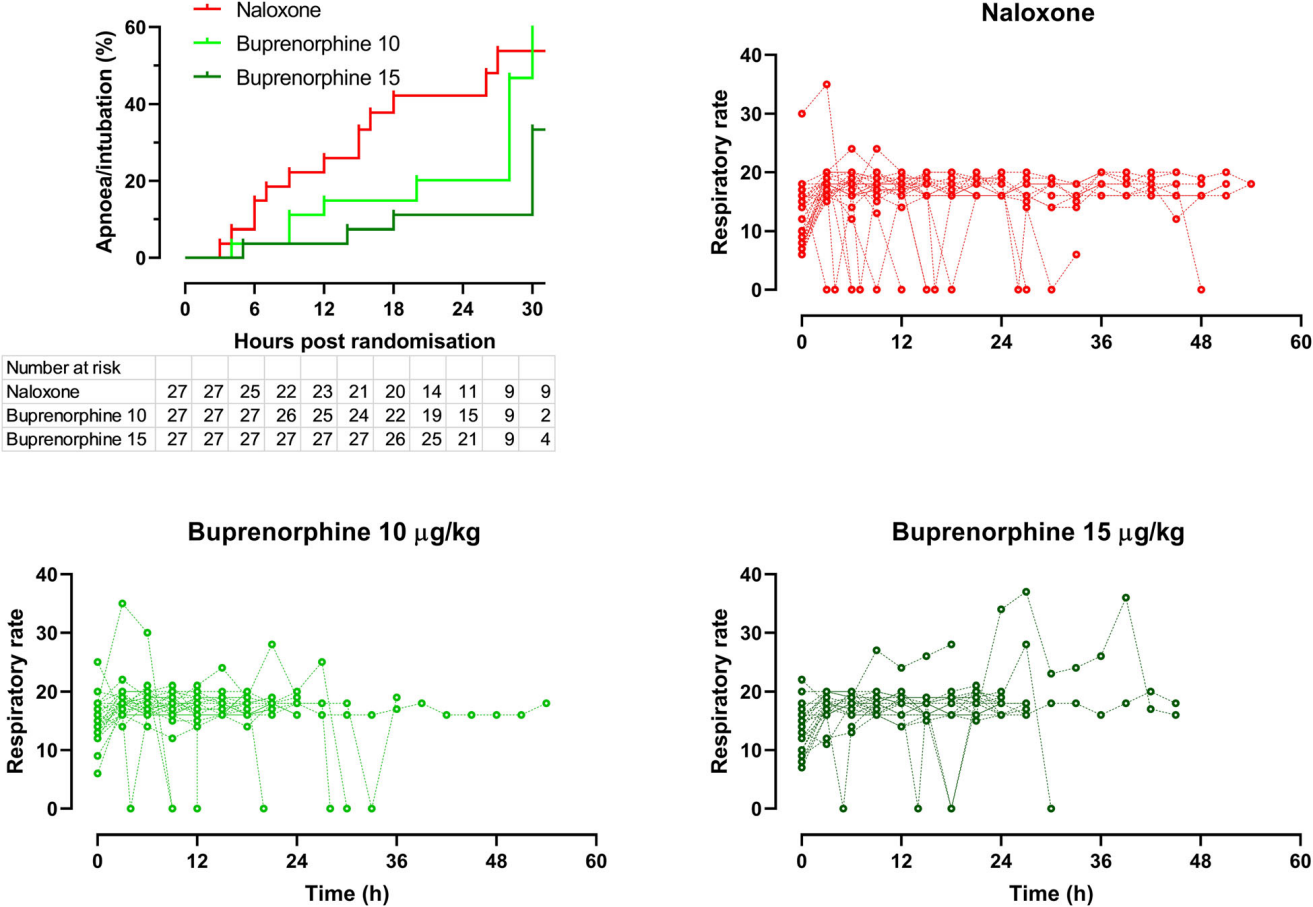
Respiratory rate over time and episodes of opioid induced apnea in those treated with buprenorphine vs naloxone (3-hourly observations + additional events). Respiratory rate shown as zero if intubated and then censored

Patients in the three treatment arms were similar in terms of their gender, age, and other variables (Table 1). Most patients responded whether they received naloxone or buprenorphine. However, complete responses were more common with buprenorphine (93% vs 48%, difference 45%, 95% CI 25 to 67%, *P* < 0.0001). Recurrence of respiratory depression was also less likely with buprenorphine (Fig. 2, combined buprenorphine vs naloxone, Mantel Cox Log rank test *χ*^2^ − 4.8, *P* = 0.0285).

**Table 1:** Demographic and baseline clinical variables in the three treatment arms

Variable †	Naloxone (*n* = 27)	Buprenorphine 10 μg/kg (*n* = 27)	Buprenorphine 15 μg/kg (*n* = 27)
Mean [IQR] Age (years) (min, max)	40 [24 to 50] (18, 66)	44 [27 to 53] (15, 63)	48 [36 to 62] (23, 77)
Mean [IQR] Methadone dose (mg)* (min, max)	100 [50 to 200](20, 350)	150 [79 to 325] (40, 1400)	100 [40 to 175] (25, 800)
Formulation: syrup	18 (67%)	19 (70%)	23 (85%)
Tablet	5 (19%)	6 (22%)	3 (11%)
Unknown	4 (15%)	2 (8%)	1 (4%)
Mean [IQR] Glasgow coma score (min, max)	14 [12 to 15] (6, 15)	15 [13 to 15] (10, 15)	13 [11 to 15] (8, 15)
Mean [IQR] Respiratory rate (min, max)	12 [8 to 16] (6, 30)	15 [14 to 18] (6, 25)	15 [10 to 16] (7, 22)
Mean [IQR] Initial naloxone dose (min, max)(*n* = 16, 19, 18)	1.0 [0.8 to 2.3] (0.4, 4.8)	0.8 [0.8 to 1.2] (0.4, 2.4)	1 [0.7 to 2] (0.4, 2)
Mean [IQR] Time to admission (h)* (min, max)	9 [5 to 12] (2, 24)	12 [7 to 20] (2, 72)	5 [5 to 12] (2, 24)
Mean [IQR] Initial pH (min, max)	7.3 [7.2 to 7.3] (7.1, 7.5)	7.3 [7.2 to 7.3] (7.1, 7.4)	7.3 [7.2 to 7.3] (7.1, 7.4)
Mean [IQR] Initial pCO_2_ (mmHg) (min, max)	56 [46 to 62] (16, 75)	57 [51 to 65] (45, 79)	62 [52 to 68] (20, 88)

† All values: Median [IQR] (min, max) unless otherwise specified. *Missing data in 27 cases

**Fig. 3:**
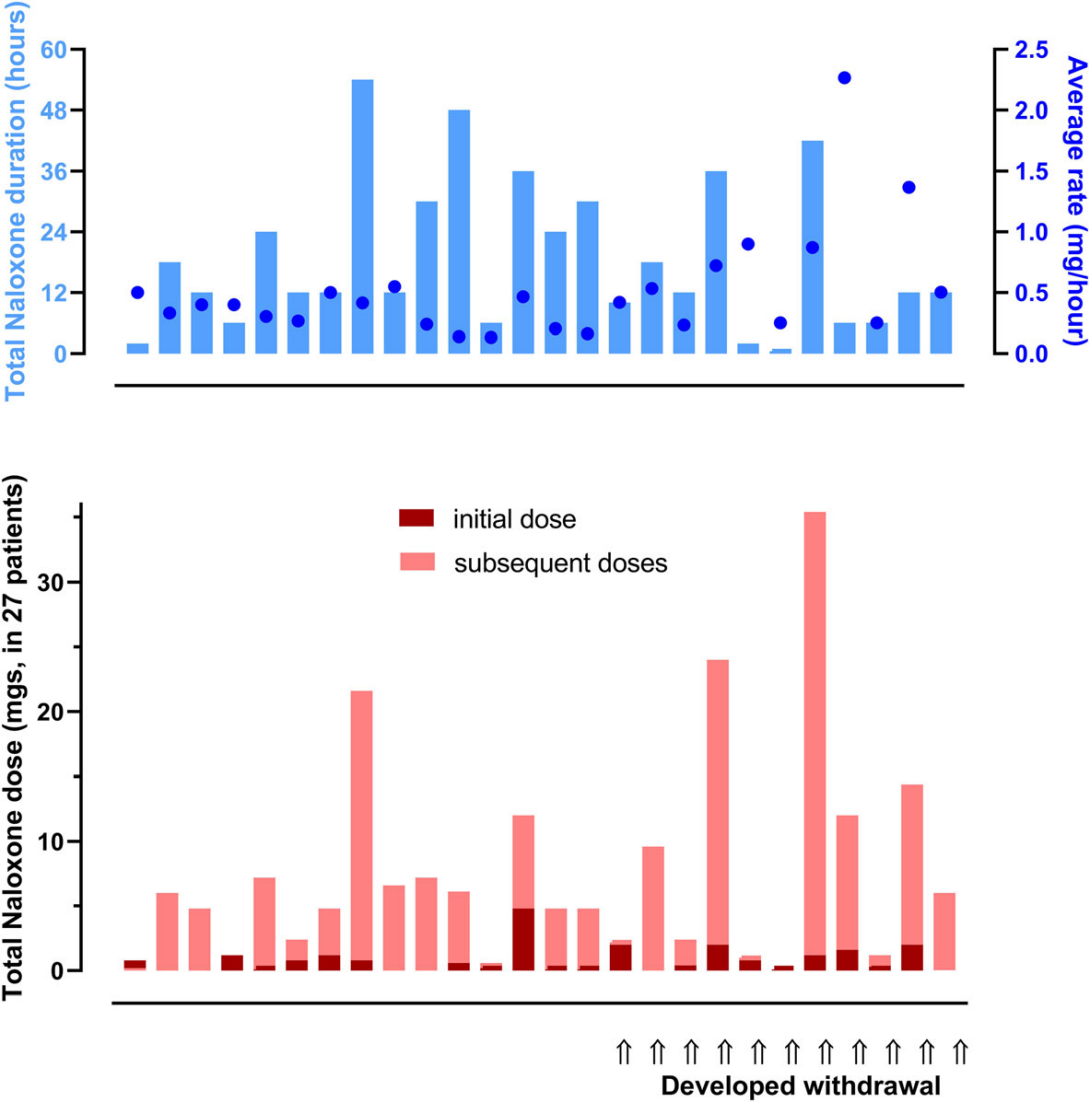
Variability in naloxone doses given to reverse opioid-induced sedation after methadone overdose

**Fig. 4:**
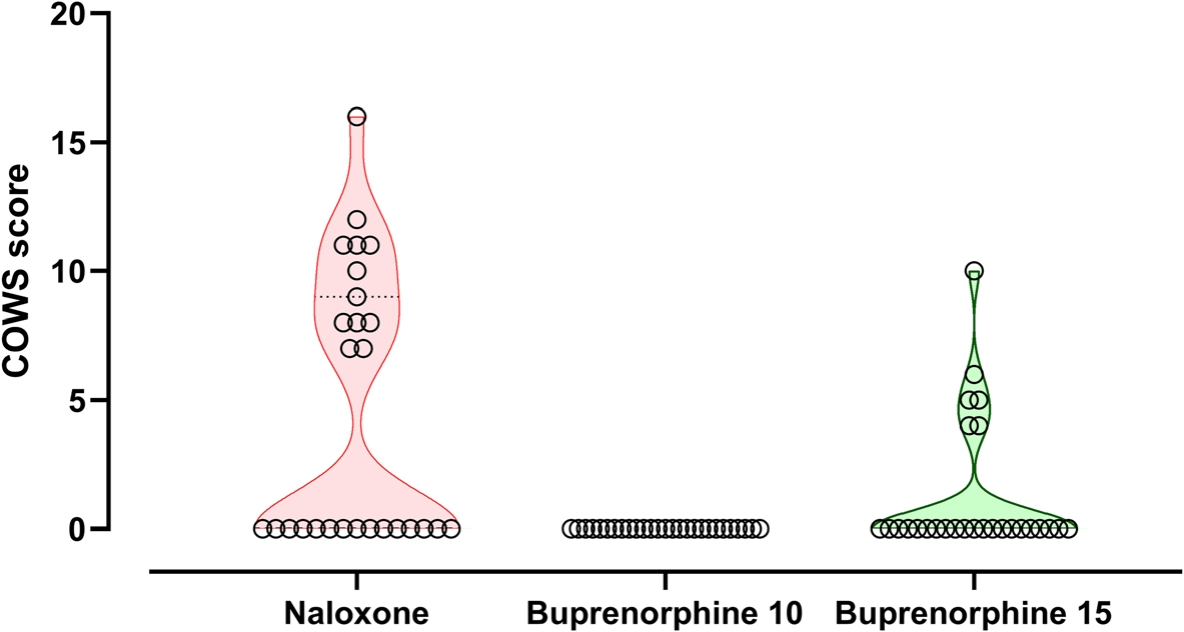
Violin and scatter plot of withdrawal scores after initial doses of buprenorphine vs naloxone (COWS scale)

**Fig. 5:**
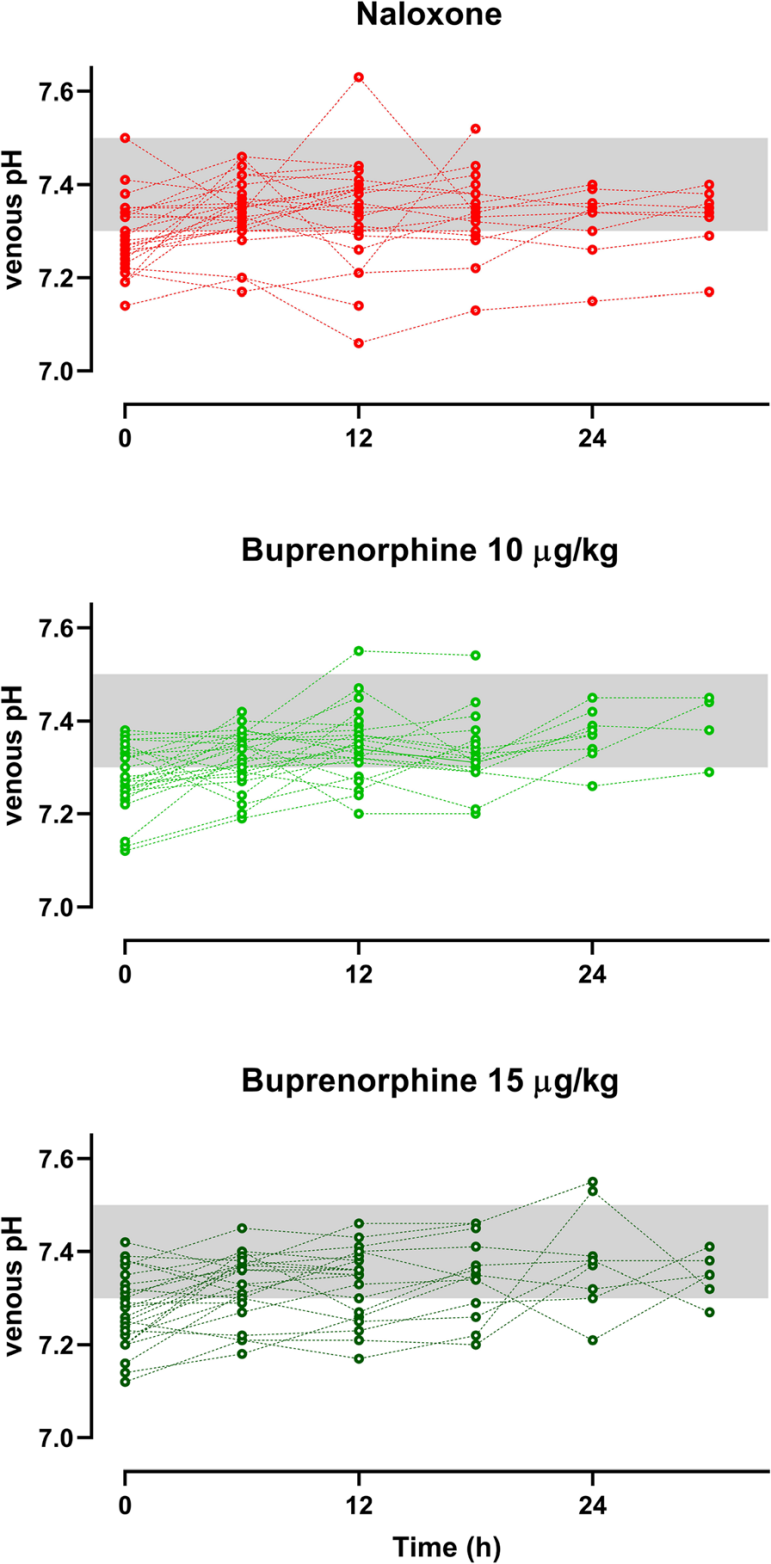
Time course and variability of venous pH after methadone overdose treated with buprenorphine vs. naloxone reversal

Patients in the naloxone arm had very variable initial doses, infusion doses, and total duration and quite commonly experienced withdrawal (Fig. 3). Risk of intubation and development of ARDS were less frequent in patients treated with buprenorphine (Table 2, Fig. 2). Withdrawal syndromes were less common and severe (Fig. 4); the duration of withdrawal signs and symptoms was not noticeably longer after buprenorphine-induced withdrawal (despite the much longer half-life). The serial blood gas data supports the consistency of response showing a more consistent PCO_2_ in the buprenorphine-treated groups (Fig. 5). The length of time in hospital was significantly shorter after buprenorphine. There were three deaths and one neurological sequela from hypoxic brain damage, all occurring in the naloxone group. The complications leading to death were ARDS, acute myocardial infarction, and multi-organ failure in the three non-survivors.

**Table 2:** Comparison of response to naloxone vs combined buprenorphine groups (*n* = 81)

Outcome	Naloxone (*n* = 27)	Buprenorphine (*n* = 54)	*P* value
Response to bolus antidote doses	Complete 13 (48%) Partial 13 (48%) No response 1(4%)	Complete 50 (93%) Partial 3 (5%) No response 1 (2%)	< 0.0001
Opioid withdrawal	15 (56%)	6 (11%)	< 0.0001
Further apnea	6 (22%)	7 (13%)	0.34
Aspiration	1 (4%)	6 (11%)	0.41
Intubation	8 (30%)	5 (9%)	0.026
Continuing Sedation	9 (33%)	3 (6%)	0.002
ARDS	4 (15%)	0	0.01
Discharged alive with no sequelae (%)	23 (85%)	54 (100%)	0.01

The higher- and lower-dose buprenorphine groups had similar outcomes; as expected, withdrawal appeared more commonly with higher doses, while re-apnea was less common and duration of action was longer in this group (Table 3). Figures 2 and 5 also illustrate these modest differences, comparing effects over time.

**Table 3:** Per-protocol comparison of outcomes in three arms of the study (*n* = 81)

Outcome	Naloxone (*n* = 27)	Buprenorphine 10 μg/kg (*n* = 27)	Buprenorphine 15 μg/kg (*n* = 27)
Response to bolus antidote doses	Complete 13 (48%) Partial 13 (48%) No response 1 (4%)	Complete 23 (85%) Relative 3 (11%) No response 1 (4%)	Complete 27 (100%) Partial 0 No response 0
Opioid withdrawal	15 (56%)	0	6 (22%)
Further apnea	6 (22%)	4 (15%)	3 (11%)
Aspiration	1 (4%)	5 (18%)	1 (4%)
Intubation	8 (30%)	4 (15%)	1 (4%)
Continuing sedation	9 (33%)	0	3 (11%)

## Discussion

In this pilot phase II study, we have shown that buprenorphine appears to be a useful antidote for methadone-induced respiratory depression. The study also highlights several potential advantages over naloxone. The dosing strategy is much simpler, the response more consistent, severe withdrawal is much less common, and the duration of action is much longer. While this study focused on methadone poisoning, this strategy has potential usefulness for all long-acting opioid overdoses in opioid-tolerant individuals. Buprenorphine, being a long-acting partial agonist, behaved as expected, reversing over-sedation without precipitating severe withdrawal.

The problems from naloxone-induced withdrawal have been highlighted in various case series of heroin overdose [4]. These problems are compounded in those ingesting longer-acting agents such as methadone [5]. Two of the deaths in the naloxone arm might have been contributed to by severe acute withdrawal, one developed respiratory complications following agitation and another a myocardial infarction. The previous human evidence on buprenorphine use as an antidote is very limited [6–8]. However, there is very considerable evidence from volunteers and in analgesia that it has much less liability to induce withdrawal than naloxone [12, 18]. It also appears to have a ceiling effect for causing respiratory depression in those naïve to opioids [14, 19].

### Limitations

This was an unfunded pragmatic clinical trial, and there are several important limitations. The sample size is typical of phase II studies using continuous outcomes. It was determined in advance in discussion with the ethics committee, but not based on statistical considerations. The method of randomization was sub-optimal and the trial was not blinded. It is possible that biases were introduced, although the baseline characteristics of the patients appeared similar and the differences seen in subjective outcomes were concordant with those that were more objective. As usual in phase II studies, we focused our analysis on the per-protocol analysis, as we wished to explore treatment effect in a tightly defined target population with methadone overdose. Our intention to treat analysis including the additional four randomized patients found similar differences between groups (see Additional file 1). The difficulties with using naloxone infusions were very apparent in our center. Some problems with the control intervention may relate to limited staffing and resources, and naloxone may produce better outcomes in other critical care settings. Further, both antagonists are likely to be less effective in mixed drug overdose, and it is also possible that the higher efficacy and low risk of withdrawal with buprenorphine might be less apparent with other opioids. Higher or lower or titrated or repeated doses of buprenorphine may be preferred strategies. Further studies are required to explore buprenorphine doses and use in other settings.

## Conclusion

Buprenorphine was an effective and safe substitute for naloxone in opioid-dependent patients who have methadone overdose and respiratory depression. It is likely to be similarly useful for overdose with other long-acting opioids. Further studies are warranted to determine the optimal dosing strategy for buprenorphine that can consistently reverse respiratory depression without precipitating withdrawal across varied overdoses and settings. Out-of-hospital buprenorphine administration to acutely overdosed patients by the patients’ family or friends might be a viable alternative to take-home naloxone.

## Additional file

Additional file 1. Comparison of response to Naloxone vs combined Buprenorphine Groups and Figure of Episodes of opioid induced apnea or intubation in those treated with buprenorphine vs naloxone Description of data: The “intention to treat” analysis is shown here comparing recurrence of respiratory depression using Mantel Cox Log rank test in two groups of naloxone and buprenorphine.

## References

[1] Peacock A, Leung J, Larney S, Colledge S, Hickman M, Rehm J (2018). Global statistics on alcohol, tobacco and illicit drug use: 2017 status report. Addiction.

[2] Degenhardt L, Charlson F, Mathers B, Hall WD, Flaxman AD, Johns N (2014). The global epidemiology and burden of opioid dependence: results from the global burden of disease 2010 study. Addiction.

[3] Kim HK, Nelson LS (2015). Reducing the harm of opioid overdose with the safe use of naloxone : a pharmacologic review. Expert Opin Drug Saf.

[4] Sivilotti ML (2016). Flumazenil, naloxone and the ‘coma cocktail’. Br J Clin Pharmacol.

[5] Khosravi N, Zamani N, Hassanian-Moghaddam H, Ostadi A, Rahimi M, Kabir A (2017). Comparison of two naloxone regimens in opioid-dependent methadone overdosed patients: a clinical trial study. Curr Clin Pharmacol.

[6] Welsh C, Sherman SG, Tobin KE (2008). A case of heroin overdose reversed by sublingually administered buprenorphine/naloxone (Suboxone). Addiction.

[7] Yokell MA, Zaller ND, Green TC, McKenzie M, Rich JD (2012). Intravenous use of illicit buprenorphine/naloxone to reverse an acute heroin overdose. J Opioid Manag.

[8] Zamani N, Hassanian-Moghaddam H (2017). Intravenous buprenorphine: a substitute for naloxone in methadone-overdosed patients?. Ann Emerg Med.

[9] Zamani N, Hassanian-Moghaddam H, Bayat AH, Haghparast A, Shadnia S, Rahimi M (2015). Reversal of opioid overdose syndrome in morphine-dependent rats using buprenorphine. Toxicol Lett.

[10] Hassanian-Moghaddam H, Zamani N, Rahimi M, Shadnia S, Pajoumand A, Sarjami S (2014). Acute adult and adolescent poisoning in Tehran, Iran; the epidemiologic trend between 2006 and 2011. Arch Iran Med.

[11] Nelson LS, Howland MA, Hoffman RS, Howland MA, Lewin NA, Nelson LS, Goldfrank LR, 10 (2015). Opioid antagonists. Goldfrank’s toxicologic emergencies.

[12] Strain EC, Preston KL, Liebson IA, Bigelow GE (1995). Buprenorphine effects in methadone-maintained volunteers: effects at two hours after methadone. J Pharmacol Exp Ther.

[13] Huestis MA, Cone EJ, Pirnay SO, Umbricht A, Preston KL (2013). Intravenous buprenorphine and norbuprenorphine pharmacokinetics in humans. Drug Alcohol Depend.

[14] Kuhlman JJ, Lalani S, Magluilo J, Levine B, Darwin WD (1996). Human pharmacokinetics of intravenous, sublingual, and buccal buprenorphine. J Anal Toxicol.

[15] Wesson DR, Ling W (2003). The Clinical Opiate Withdrawal Scale (COWS). J Psychoactive Drugs.

[16] Fan E, Brodie D, Slutsky AS (2018). Acute Respiratory Distress Syndrome: Advances in Diagnosis and Treatment. JAMA.

[17] Whitehead AL, Julious SA, Cooper CL, Campbell MJ (2016). Estimating the sample size for a pilot randomised trial to minimise the overall trial sample size for the external pilot and main trial for a continuous outcome variable. Stat Methods Med Res.

[18] Strain EC, Preston KL, Liebson IA, Bigelow GE (1992). Acute effects of buprenorphine, hydromorphone and naloxone in methadone-maintained volunteers. J Pharmacol Exp Ther.

[19] Kalluri HV, Zhang H, Caritis SN, Venkataramanan R (2017). A physiologically based pharmacokinetic modelling approach to predict buprenorphine pharmacokinetics following intravenous and sublingual administration. Br J Clin Pharmacol.

