# Supplementary material for: Buprenorphine to reverse respiratory depression from methadone overdose in opioid-dependent patients: a prospective randomized trial
Source: Crit Care. 2020 Feb 7;24:44. doi: 10.1186/s13054-020-2740-y (PMC7006192; doi:10.1186/s13054-020-2740-y)
Supplement: Supplementary file 1 — Comparison of response to Naloxone vs combined Buprenorphine Groups and Figure of Episodes of opioid induced apnea or intubation in those treated with buprenorphine vs naloxone Description of data: The “intention to treat” analysis is shown here comparing recurrence of respiratory depression using Mantel Cox Log rank test in two groups of naloxone and buprenorphine. [file 13054_2020_2740_MOESM1_ESM.doc]

Buprenorphine to reverse respiratory depression from methadone overdose in opioid-dependent patients: A Prospective Randomized Trial The “intention to treat” analysis is shown below. There were 4 additional patients randomized who had negative urine tests for methadone. It is likely they ingested another opioid drug, but they may have had less opioid tolerance. They all had complete response to their antagonist treatment, none had evidence of withdrawal or further complications.

Complete responses were more common with buprenorphine (93% vs 48%, difference: 45%, 95% CI:25 to 67%, P<0.0001). Recurrence of respiratory depression was less likely with buprenorphine (Figure, combined buprenorphine vs naloxone, Mantel Cox Log rank test χ2 – 4.3, P=0.0379).

Table 1: Comparison of response to Naloxone vs combined Buprenorphine Groups (n=85)

| ***Outcome*** | ***Naloxone***  ***(n=29)*** | ***Buprenorphine***  ***(n=56)*** | ***P value*** |
| --- | --- | --- | --- |
| Response to bolus antidote doses | Complete 15 (52%)  Partial 13 (45%)  No response 1(3%) | Complete 52 (93%)  Partial 3 (5%)  No response 1 (2%) | <0.0001 |
| Opioid withdrawal | 15 (52%) | 6 (11%) | <0.0001 |
| Further apnea | 6 (21%) | 7 (13%) | 0.35 |
| Aspiration | 1 (3%) | 6 (11%) | 0.41 |
| Intubation | 8 (28%) | 5 (9%) | 0.052 |
| Continuing Sedation | 9 (31%) | 3 (5%) | 0.002 |
| ARDS | 4 (14%) | 0 | 0.01 |
| Discharged alive with no sequelae (%) | 23 (86%) | 54 (100%) | 0.01 |

Table 2: Comparison of patients in Buprenorphine group who were and were not pre-treated by naloxone

| ***Outcome*** | ***Naloxone Administered Before Presentation***  ***(n=37)*** | ***Naloxone not Administered Before Presentation***  ***(n=17)*** | ***P value*** |
| --- | --- | --- | --- |
| Response to buprenorphine bolus | Complete; 35 (95%)  Partial/none; 2 (5%) | Complete; 15 (88%)  Partial/none; 2 (12%) | 0.33 |
| Opioid withdrawal | Yes; 5 (13%)  No; 32 (87%) | Yes; 1 (6%)  No; 16 (94%) | 0.38 |
| Further apnea | Yes; 6 (19%)  No; 30 (81%) | Yes; 1 (6%)  No; 16 (94%) | 0.21 |
| Intubation | Yes; 3 (8%)  No; 34 (92%) | Yes; 2(12%)  No; 15 (88%) | 0.51 |
| Continuing Sedation | Yes; 2 (5%)  No; 35 (95%) | Yes; 1 (6%)  No; 16 (94%) | 0.69 |
| Aspiration | Yes; 5 (11%)  No; 33 (89%) | Yes; 1 (6%)  No; 16 (94%) | 0.49 |
|  |  |  |  |
|  |  |  |  |

**Figure. Episodes of opioid induced apnea or intubation in those treated with buprenorphine vs naloxone – ITT analysis.**
